# Effect of Adherence to Oral Semaglutide on Glycemic Control in People With Type 2 Diabetes Treated With Metformin: Protocol for an Open-Label Clinical Trial

**DOI:** 10.2196/64899

**Published:** 2025-09-23

**Authors:** Nynne Sophie Holdt-Caspersen, Claus Dethlefsen, Ole Hejlesen, Erik Christiansen, Stine Hangaard, Peter Vestergaard, Morten Hasselstrøm Jensen

**Affiliations:** 1 Department of Data Science Novo Nordisk A/S Aalborg Denmark; 2 Department of Health Science and Technology Aalborg University Aalborg Denmark; 3 Department of Mathematical Sciences Aalborg University Aalborg Denmark; 4 Department of Medical & Science Novo Nordisk A/S Søborg Denmark; 5 Steno Diabetes Center North Denmark Aalborg University Hospital Aalborg Denmark; 6 Department of Endocrinology Aalborg University Hospital Aalborg Denmark; 7 Department of Clinical Medicine Aalborg University Aalborg Denmark

**Keywords:** medication adherence, continuous glucose monitoring, observational study, oral antidiabetic drug, dosing instructions, oral semaglutide

## Abstract

**Background:**

Treatment adherence by people with type 2 diabetes (T2D) is overall suboptimal, which can hinder glycemic control. Multiple adherence barriers have been identified, such as the dislike and fear of injections. Several of the recommended antidiabetic drugs are available in oral formulations, which may be a good alternative to injection therapy when possible. However, strict dosing instruction could pose adherence barriers; for example, oral semaglutide requires predose and postdose fasting and restricted water intake at dosing time. Currently, oral semaglutide is the only oral glucagon-like peptide-1 receptor agonist and has only been available for a few years; therefore, limited knowledge exists on adherence to it.

**Objective:**

The aim of this study is to investigate the effect of adherence to oral semaglutide dosing instructions on glycemic control in people with T2D who are dysregulated on metformin and optionally a sodium-glucose cotransporter-2 inhibitor and naïve to oral semaglutide.

**Methods:**

This prospective, noninterventional, open-label, clinical trial with a duration of 12 weeks will be conducted in Denmark. Eligible participants are adults (aged ≥18 years) with dysregulated T2D (hemoglobin A1c of 53-75 mmol/mol) currently treated with metformin and optionally a sodium-glucose cotransporter-2 inhibitor for whom the next natural step in the treatment is to add an antidiabetic drug to the treatment regimen. Potential participants are recruited through announcements on social media and digital mail sent to their official digital mailbox (e-boks). During the trial, 20 participants will be initiated on oral semaglutide and escalated in dosage in accordance with the label. Information on the participants’ behavior related to the dosing instructions will be collected using the following devices: a smartwatch to track activity and sleep time, a smart pill bottle to track dosing time, a smart bottle to track time and volume of water intake at dosing time, and a smartphone to take a photo of their breakfast to log time of breakfast. Glycemic control will be assessed using an unblinded continuous glucose monitoring sensor that the participants will wear. Participants are asked to report any cases of nausea or vomiting in terms of time of occurrence, duration, and severity. The primary endpoint is change from baseline to end-of-study time-in-range derived from continuous glucose monitoring data.

**Results:**

The first participant visit was in April 2024. Three months of high frequency temporal data on adherence behavior will be collected, despite the relatively few expected participants included.

**Conclusions:**

Participants may change their behavior due to awareness of being observed. Regardless, the knowledge gained from this trial might be integrated into a decision support system, providing people with diabetes with guidance on how to increase adherence and potentially improving glycemic control.

**Trial Registration:**

ClinicalTrials.gov NCT06333080.; https://clinicaltrials.gov/study/NCT06333080

**International Registered Report Identifier (IRRID):**

DERR1-10.2196/64899

## Introduction

In the adult population worldwide, 10.5% had a diabetes diagnosis in 2021, and an increase in prevalence is expected [1]. A profound majority of people with diabetes, approximately 91%, have type 2 diabetes (T2D). [2]. The goal in diabetes management is to control blood glucose levels to decrease the risk of developing long-term complications [3]. The recommended glycated hemoglobin (HbA1c) level is ≤53 mmol/mol in nonpregnant adults, but the target blood glucose level should be individualized based on each patient according to the American Diabetes Association [3].

The initial antidiabetic drug used is often metformin, although the first-line treatment can be other antidiabetic drugs or a combination of antidiabetic drugs, especially for patients with severe hyperglycemia or some renal and cardiovascular diseases [4]. As T2D progresses, the initial treatment may fail to maintain glycemic control and is therefore often supplemented [4]. The recommended choices of supplemental medication in people with T2D without other comorbidities are glucagon-like peptide-1 (GLP-1) receptor agonists (RAs), dual glucose-dependent insulinotropic polypeptide (GIP) and GLP-1 RAs, sodium-glucose cotransporter-2 inhibitors (SGLT2i), dipeptidyl peptidase-4 inhibitors, sulfonylurea, and thiazolidinedione [4]. According to the American Diabetes Association [4], the choice of antidiabetic drugs should consider both glycemic and weight management goals along with comorbidities, adverse events, and the patient’s preferences.

Adherence to the antidiabetic treatment has been reported to be suboptimal [5-8], reducing the effect of the drugs. The patients’ preferences on the side effect profile, perceived complexity of the treatment, and the route of administration may all be barriers to good adherence [9-11]. Injection therapy can be associated with discomfort and fear of injection together with the feeling of stigmatization, thus leading to nonadherence [12]. This specific barrier can be removed by choosing a treatment regimen consisting entirely of oral antidiabetic drugs (OADs) for as long as possible based on the recommendations of the American Diabetes Association. As all but the dual GLP-1/GIP RA and insulin are available in an oral formulation, these are potential alternatives to the injection therapies.

The oral formulation of GLP-1 RA, oral semaglutide, has shown promising results for reducing HbA1c and body weight [13]. However, the dosing complexity of oral semaglutide may be perceived as high, as the intake of food both before and after intake of the drug decreases the exposure of semaglutide [14]. In a food-effect study, no or limited semaglutide exposure was observed in participants who had eaten prior to the intake of semaglutide [14]. Similarly, a study on dosing conditions found that food intake shortly after the intake of oral semaglutide decreased semaglutide exposure, whereas a longer duration of fasting resulted in greater exposure [14]. In addition, exposure of semaglutide is affected by the water volume administered with the oral semaglutide [14,15]. These studies resulted in rather complex dosing instructions, namely that oral semaglutide should be administered in a fasting state with up to 120 mL water followed by a postdose fasting period of minimum 30 minutes with no intake of liquids or other medications [16,17]. Furthermore, gastrointestinal adverse events, such as nausea and vomiting, are associated with semaglutide. The occurrence of gastrointestinal adverse events is reported to be most frequent at the time of dose escalation [18]. The rather strict dosing instructions of oral semaglutide combined with the potential occurrence of gastrointestinal adverse events may present a barrier for adhering to the dosing instructions. Poor adherence may result in decreased semaglutide exposure, which may lead to reduced glycemic control.

Knowledge on adherence to oral semaglutide is limited as it is the first and only marketed oral GLP-1 RA and has only been available on the market for a few years [16,17]. Furthermore, adherence to oral semaglutide is not necessarily similar to that of other classes of OADs, as adherence across these drug classes has been found to differ [19]. Thus, a clinical trial investigating the effect of adherence to oral semaglutide dosing instructions on glycemic control is warranted, and to the best of our knowledge, no studies have collected continuous glucose monitoring (CGM) and high frequency temporal data on behavioral patterns of people with T2D on oral semaglutide. Previous reviews on the adherence to OADs, including by the authors of this paper, have based the adherence measurements on pharmacy claims [5-8]. Therefore, it is not possible to ascertain how the adherence behavior of the person with diabetes affects their glycemic level based on existing evidence. An investigation of the effect of adherence to the oral semaglutide dosing instructions on glycemic control, including the influence of side effects, could provide this new knowledge. Thus, the objective of the present trial is to investigate the effect of adherence to oral semaglutide dosing instructions on glycemic control in people with T2D who are dysregulated on metformin and optionally an SGLT2i and naïve to oral semaglutide.

## Methods

### Setting and Design of the Study

The study will be conducted at Steno Diabetes Center North Denmark (SDCN) at Aalborg University Hospital, Aalborg, Denmark. This study protocol was based on the SPIRIT (Standard Protocol Items: Recommendations for Interventional Trials) guidelines [20].

**Figure 1:**
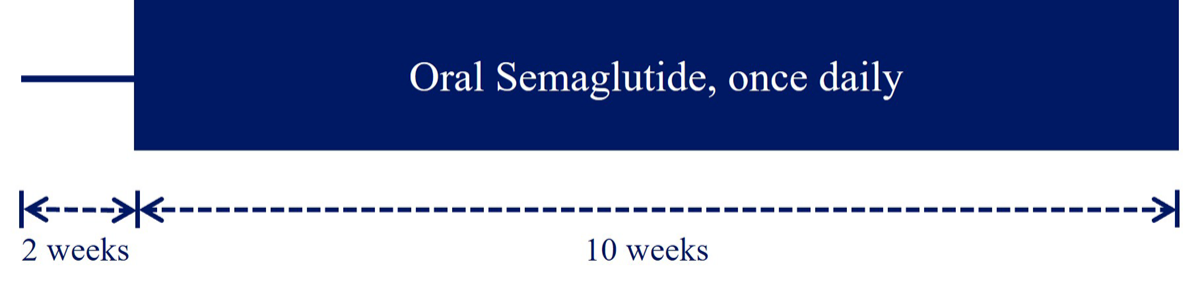
Study design of the clinical trial. The total duration of the trial is 12 weeks, with oral semaglutide being initiated after the second week of the trial.

The trial is a prospective, noninterventional, open-label, clinical trial. As the trial is observational, neither the project group nor the participants will be blinded. The duration of the trial is 12 weeks, during which participants with dysregulated T2D on metformin and optionally an SGLT2i will continue their current treatment, and treatment with oral semaglutide will be initiated 2 weeks after the trial begins (Figure 1).

### Selection and Screening of Participants

#### Eligibility Criteria

A total of 20 participants meeting the eligibility criteria will be included in the trial. Eligible trial participants are adults ( aged ≥18 years) who for at least 1 year have had a diagnosis of T2D and have been in treatment with metformin and optionally an SGLT2i. A participant will only be eligible if a HbA1c of 53-75 mmol/mol has been recorded less than 3 months prior to trial inclusion. As the participants are not in glycemic control, the next step in the treatment is to add an antidiabetic drug to obtain glycemic control [4], which in this clinical trial is oral semaglutide.

Thus, eligible participants for this study should have an antidiabetic drug added to their current treatment regardless of participation in this study. The participants will continue their current treatment, and treatment with oral semaglutide will be initiated 3 weeks after inclusion in the trial. Furthermore, the participants must be able to understand and read Danish and be able to use a smartphone as well as the devices used in the clinical trial.

Potential participants will be excluded if they previously have received treatment with glucose-lowering medications other than metformin and SGLT2i. Potential participants who are only treated with metformin and have cardiovascular or kidney disease, which would indicate use of other second-line treatments such as SGLT2 inhibitors or subcutaneous GLP1 receptor agonists, are not eligible for inclusion. Participants will also be excluded if any of the following apply: (1) they have other types of diabetes than T2D, (2) they are participating in an interventional trial, (3) they are pregnant or breastfeeding, (4) they have known retinopathy, (5) they have a known allergy to semaglutide, (6) they have major surgery planned during the trial period, (7) they have uncontrolled cancer, (8) they have a personal or family history of medullary thyroid carcinoma, (9) they have multiple endocrine neoplasia syndrome type 2, or (10) they have other conditions that the investigators deem to render the participant unfit for inclusion in the trial, including a physical or cognitive inability to participate in the trial.

#### Recruitment

Trial participants will be recruited using the following different strategies: (1) telephone calls to a group of potential participants who have agreed to be contacted regarding the initiation of new trials at SDCN; (2) announcements in newspapers, the Danish diabetes magazine, the website of Region North Denmark, social media, and relevant patient organizations; (3) posters and leaflets placed at SDCN, Aalborg University Hospital, and other relevant health centers; and (4) digital mail sent to the official digital mailbox (e-boks) of a list of potential participants (people with T2D on metformin and optionally SGLT2i with high HbA1c obtained less than 3 months ago), which will be obtained from the Business Intelligence Unit at the North Denmark Region.

#### Informed Consent

A participation information letter describing the trial will be mailed or handed to interested potential participants. An information interview will be held at Aalborg University Hospital with the interested potential participant, an optional companion, and either the investigator, subinvestigator, or a delegated project member with the required professional knowledge. In the information interview, the potential participant will be informed on the voluntary nature of participating in the trial, their right to reflect on whether to participate prior to giving informed consent to trial participation, and their right to withdraw consent at any time, without any explanation. A participant cannot be included in the trial until informed consent has been signed by both the participant and the investigator.

### Data Collection

#### Trial Flow

**Figure 2:**
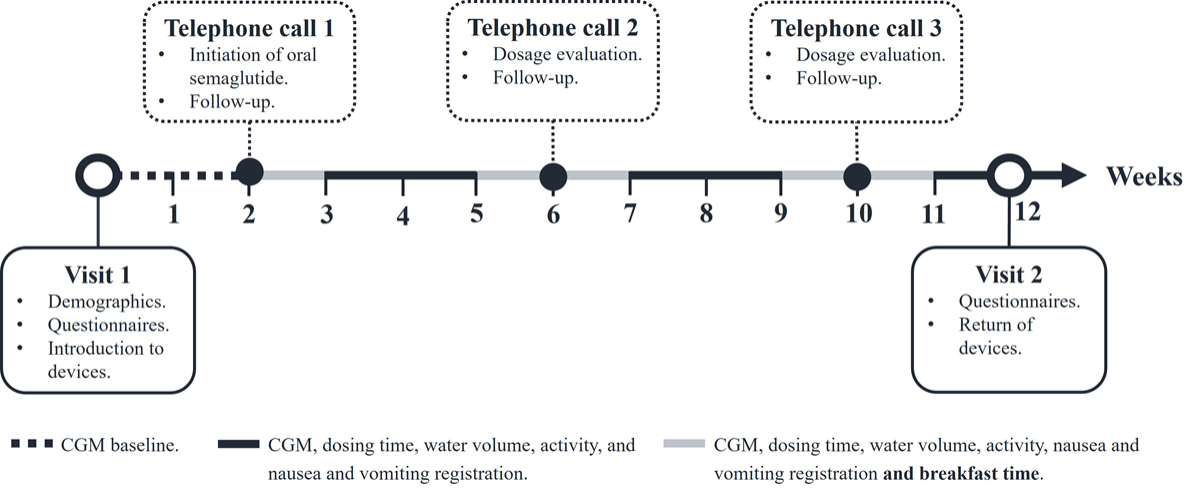
Trial flowchart for each participant. CGM: continuous glucose monitoring.

The trial period starts with the first visit to the trial site and ends 12 weeks later with the second visit to the trial site. A CGM baseline reading from each participant will be collected prior to oral semaglutide initiation, corresponding to the first 2 weeks of the trial period. After oral semaglutide initiation, CGM data, physical activity, time of dosing, and water volume intake at dosing time will be collected throughout the remaining trial period. The participants will be asked to report occurrences of nausea and vomiting, including time, duration, and severity (using a scale from 0-10 with 10 being the worst imaginable) in a paper diary. In addition, the participants will be asked to register the time of breakfast at 3 time periods during the trial (Figure 2).

All participants will continue their current treatment throughout the trial period. Additionally, the participants will be started on oral semaglutide 2 weeks after trial initiation, at which time a lab technician from SDCN will contact the participant by phone (Figure 2). The oral semaglutide will be handed out to the participants at the first visit to the trial site. The initiation and dosage evaluation of oral semaglutide will be in accordance with the product information on dose escalation [16,17]. Thus, the initial dosage of oral semaglutide will be one 3 mg tablet once daily (OD), which the participants will be instructed to take in the morning in a fasting state with maximum of a half glass of water, approximately 120 mL. Participants will be instructed to wait at least 30 minutes before the intake of food, liquids, or other medications. After 4 weeks, corresponding to telephone call 2 (Figure 2), the dosage of oral semaglutide will be increased to one 7 mg tablet OD [16,17]. After an additional 4 weeks, corresponding to telephone call 3 (Figure 2), the dosage of oral semaglutide will be re-evaluated and, if needed, increased to one 14 mg tablet OD to reach the glycemic target of the participant [16,17]. The dosage evaluation will be based on the collected CGM data, which the trial staff at SDCN will access remotely in connection with telephone call 3.

#### Demographic Data

Demographic information on each participant will be obtained at the first visit and includes birth date, biological sex, ethnicity, height and body weight, systolic and diastolic blood pressure, duration of diabetes (defined as the time from T2D diagnosis), diabetes complications, smoking status, alcohol intake, exercise, diet, marital status, housing status, level of education, employment status, and concomitant medications and illnesses.

#### Questionnaires

The participants’ view on their adherence to the treatment and perceived adherence barriers will be captured using previously published questionnaires. Data on health beliefs will be obtained by asking the participants to answer the questionnaire developed by Given et al [21] and adapted by Becker and Janz [22]. The participants will also be asked to answer a questionnaire on social support developed by Sarason et al [23].

**Figure 3:**
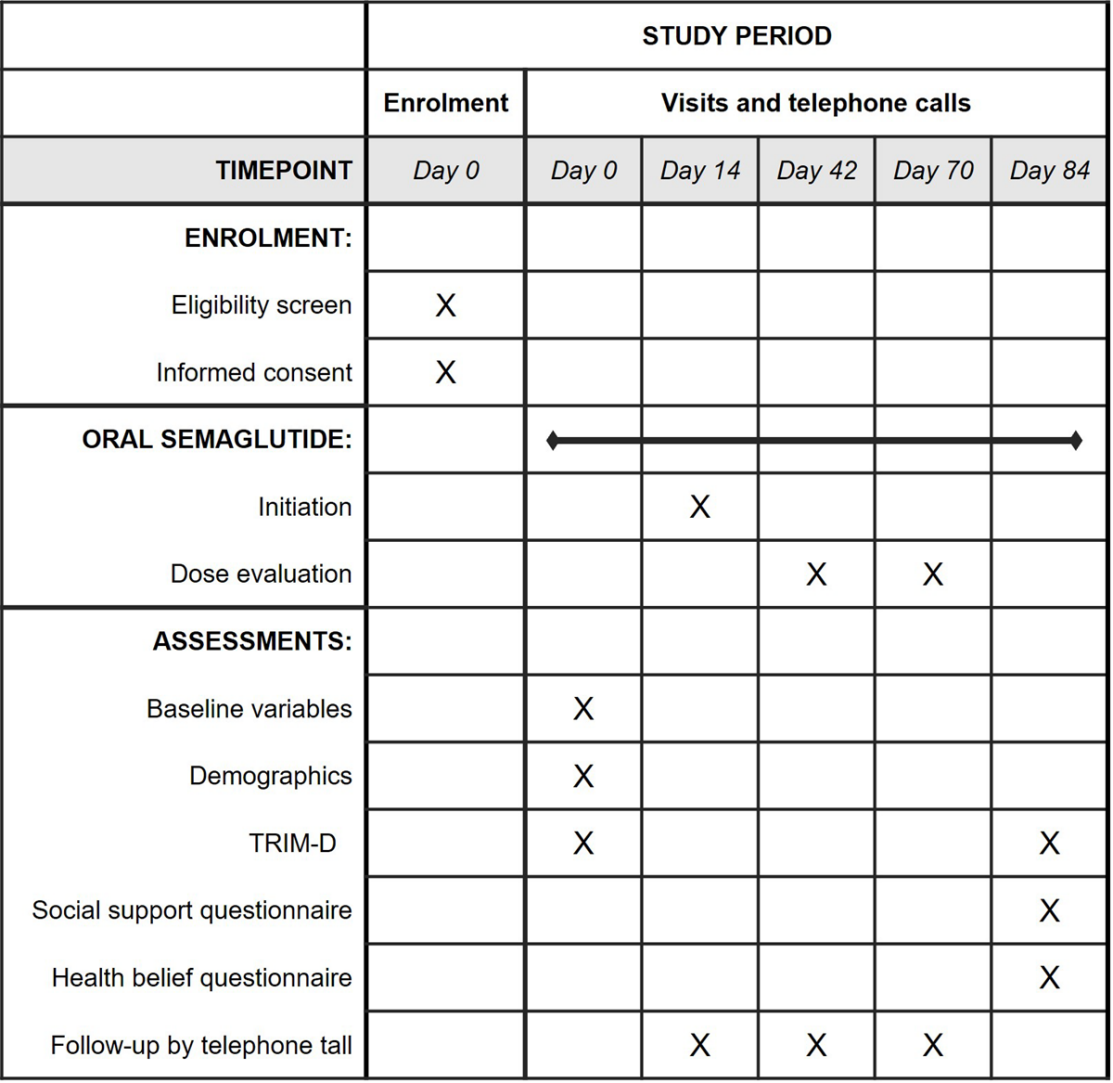
Schematic overview of participant enrolment, initiation and escalation of oral semaglutide, participation follow-up by telephone, and assessment of baseline variables, demographics, and questionnaires. The schematic overview is based on the SPIRIT (Standard Protocol Items: Recommendations for Interventional Trials) guidelines. TRIM-D: treatment-related impact measure for diabetes.

The patient-reported adherence to and satisfaction with the diabetes treatment will be assessed using the treatment-related impact measure for diabetes (TRIM-D) questionnaire, which consists of 5 subcategories: treatment burden, daily life, diabetes management, compliance, and psychological health [24]. The participants will be asked to answer the TRIM-D questionnaire at the start and end of the trial. Figure 3 provides a schematic of the trial timeline.

#### Devices

Throughout the trial, data will be collected by asking the participants to use or wear several devices to collect data. These devices include an unblinded CGM sensor (Dexcom G6 CGM System, Dexcom Inc) to determine the level of glycemic control, a smartwatch (Fitbit Charge 4, Fitbit Inc) worn all hours of the day to track activity and determine time of sleep, a smart pill bottle (MEMS Cap Smart Pill Bottle, Aardex Group) to collect time of dosing, a smart bottle (HidrateSpark Pro, HidrateSpark) to collect time and volume of water intake at dosing time, and a smartphone to take a photo of their breakfast in the week before and after dosage evaluation (Figure 2) to collect time of breakfast data.

### Discontinuation Criteria and Adverse Events

#### Discontinuation From Trial

Completion of the trial is defined as attendance at the final visit regardless of the degree to which the devices of the trial have been used. The entire trial is complete after the final visit by the last participant. In case a participant wants to withdraw from the trial, a visit at the trial site will be offered. All questions the participant may have will be answered and the devices returned.

A participant will be withdrawn from the trial if the participant experiences severe hypoglycemia or hyperglycemia or ketoacidosis that the investigators attribute to the trial, or if the investigators deem the participant unfit to continue the trial. The entire trial will be stopped prematurely if serious adverse events associated with the trial, as judged by the principal investigator or subinvestigators, occur.

#### Adverse Events

The disadvantages of participation are considered to be limited. These disadvantages include the inconvenience of trial participation and use of the CGM sensor, which may cause local skin irritation and elevates the risk of infection. As oral semaglutide will be initiated in the trial, side effects due to oral semaglutide may occur. The most frequently reported side effects of oral semaglutide are related to the gastrointestinal symptoms, such as nausea, vomiting, diarrhea, constipation, stomach pain, gastritis, decreased appetite, and gastroesophageal reflux disease [16,17]. Other common side effects include tiredness, hypoglycemia, and complications of retinopathy [16,17]. Uncommon side effects are gall stones, weight loss, and fast pulse [16,17]. Rare side effects include acute pancreatitis and anaphylactic reaction [16,17].

Adverse events occurring during the trial period will be reported, and the investigators will note whether the adverse event in their opinion is related to trial participation in accordance with the guidelines of the National Committee on Health Research Ethics.

### Outcome Measures

#### Primary Endpoint

The primary endpoint is the change from baseline to end-of-study time-in-range (TIR) derived from CGM data calculated in accordance with the International Consensus on Use of Continuous Glucose Monitoring [25].

#### Secondary Endpoints

Secondary endpoints include the following:

1. Time elapsed from last food intake to dosing time.
2. Time elapsed from dosing time to time of breakfast.
3. Occurrence of nausea or vomiting, including timing, duration, and severity. Nausea and vomiting severity will be reported on a scale from 0-10, with 0 being no nausea or vomiting and 10 being the worst imaginable nausea or vomiting, similar to the scale of nausea severity described by Leere et al [26].
4. Volume of water intake at dosing time.
5. Patient-reported satisfaction and adherence to treatment reported as the total score of the TRIM-D questionnaire.

#### Exploratory Endpoints

An exploratory endpoint is steps per day. Additionally, qualitative interviews will be carried out with selected participants to explore their adherence to oral semaglutide during the trial period.

#### Statistical Considerations

Statistical analyses will be performed on the data of all included trial participants, and a statistical significance level of 5% (α=.05) will be used.

#### Primary Analysis

The primary endpoint (change from baseline to end-of-study in TIR) will be analyzed using a simple linear regression model with the average (in weeks 10-12) of time elapsed from dosing time to time of breakfast.

#### Sample Size

With 20 participants, there is 80% power to detect a significant correlation of 0.58 between the elapsed time from dosing to time of breakfast and the improvement in TIR.

#### Missing or Unused Data

CGM-derived metrics such as TIR are only calculated for a specific interval if data have been collected ≥80% of the time in accordance with the International Consensus on Use of Continuous Glucose Monitoring [25]. Missing data will be treated as missing at random, and multiple imputation to impute missing data will be used in the statistical analysis.

### Data Management

#### Biological Material

No biological material will be obtained from the trial participants.

#### Handling and Archiving Data

Data storage will consist of both paper files and digital files on a computer using Research Electronic Data Capture (REDCap) [27], both of which will be accessible only by the investigator, subinvestigators, and relevant members of the project team. Data related to the trial will be kept stored for the duration reported to the Danish Data Protection Agency. Third-party data use will only occur if agreed upon by the subinvestigators and the primary investigator.

The electronic journal of potential trial participants will be accessed by health care professionals affiliated with SDCN to assess if the potential participant is suitable for trial inclusion by reviewing information including health status, medications, and comorbidities. The electronic journal will not be accessed without the participants’ consent. Furthermore, prospective inspection authorities will be given access to the patients’ electronic journal in relation to monitoring and quality control of the trial.

### Ethical Considerations

The trial has been approved by the Regional Committee on Health Research Ethics of the North Denmark Region (N-20220017) and will be conducted in compliance with the good clinical practice guidelines and the Helsinki declaration. In case changes to the trial protocol are needed, an amendment will be submitted to the Regional Committee on Health Research Ethics of the North Denmark Region for approval. No publications related to this trial will contain information that can identify the trial participants. If future studies based on the data collected in this trial require additional consent from the participants, they will be contacted before any such studies are undertaken. Authorship will be granted based on the Vancouver recommendations [28].

## Results

This trial protocol is version 6 and was approved on August 12, 2024. The first participant visit was on April 16, 2024.

## Discussion

The present clinical trial will be conducted with the objective of investigating the effect of adherence to oral semaglutide dosing instructions on glycemic control in people with T2D who are dysregulated on metformin and optionally an SGLT2i and naïve to oral semaglutide. Several previous studies investigating adherence to various OADs based their findings on pharmacy claims data [5-8]. Pharmacy claims data are limited in that it is not possible to know if the medication was administered as per the dosing instructions or at all, and the reasons for potential nonadherence or changes in medication type are unknown.

The main limitation of this study is the relatively low number of trial participants that are planned to be included. However, as high frequency temporal data will be collected for each participant over a 3-month period, each participant will act as their own control over the trial period.

A further limitation of this study is that participants might change their behavior due to their awareness of being observed [29], thus inducing noise in the data. The relatively large number of devices the participants will be asked to use during the trial could lead to the participants feeling burdened, especially participants with low digital literacy, which could result in reduced use of these devices and lowered data quality. This burden has been lowered by asking participants to take photos only of their breakfast and only at certain times of the trial. Additionally, an inclusion criterium of the trial is that the participant is able to use a smartphone and the devices of the trial.

In a study by Babazadeh et al [30], selfcare behaviors such as adherence to antidiabetic drugs and attention to diet were found to affect HbA1c levels in people with T2D. Likewise, studies by Ranjbaran et al [31] and Döbler et al [32] investigated interventions to increase adherence to diet and medication. In both studies, an improvement in HbA1c was observed for the intervention group compared to the control group [31,32], suggesting that delivering these types of interventions can be beneficial for people with T2D to achieve greater adherence.

The knowledge gained from the current trial could be integrated into a decision support system, providing people with T2D with guidance on how to increase their adherence and potentially improve glycemic control. Such a decision support system could, for example, provide people with T2D with a visualization of glycemic control in response to their adherence and automated guidance on how to further increase adherence. Information on glycemic control can be obtained using CGM and the level of adherence can be determined by collecting data on behavioral patterns influencing adherence, such as predose and postdose fasting, water intake at dose time, adverse events, and physical activity, depending on the findings of this clinical trial.

## Abbreviations

CGM: continuous glucose monitoring
GIP: glucose-dependent insulinotropic polypeptide
GLP-1: glucagon-like peptide-1
HbA1c: glycated hemoglobin A1c
OAD: oral antidiabetic drug
OD: once daily
RA: receptor agonist
SDCN: Steno Diabetes Center North Denmark
SGLT2i: sodium-glucose cotransporter-2 inhibitor
SPIRIT: Standard Protocol Items: Recommendations for Interventional Trials
T2D: type 2 diabetes
TIR: time-in-range
TRIM-D: treatment-related impact measure for diabetes

## Data Availability

Data related to the trial will be only accessible by the investigator, subinvestigators, and relevant members of the project team and will be kept stored for the duration reported to the Danish Data Protection Agency. Third-party data use will only occur if agreed upon by the subinvestigators and the primary investigator. The electronic journals of potential and included trial participants will solely be accessed by health care professionals affiliated with the SDCN and only after the patients have provided consent. Prospective inspection authorities will be given access to the patients’ electronic journal in relation to monitoring and quality control of the trial.
